# Latent variable modelling of condition and reproductive investment in *Gryllus vocalis* field crickets

**DOI:** 10.1098/rsos.261085

**Published:** 2026-10-28

**Authors:** Madison Von Deylen, Agustí Muñoz-Garcia, Ian M. Hamilton, Susan N. Gershman

**Affiliations:** ^1^ Evolution, Ecology, and Organismal Biology, The Ohio State University, Columbus, OH, USA; ^2^ Evolution, Ecology, and Organismal Biology, The Ohio State University at Mansfield, Mansfield, OH, USA; ^3^ Mathematics, The Ohio State University, Columbus, OH, USA; ^4^ Evolution, Ecology, and Organismal Biology, The Ohio State University at Marion, Marion, OH, USA

**Keywords:** multivariate analysis, fitness, life history, resource allocation

## Abstract

‘Condition’ is often used to describe the underlying state or quality of an individual and is expected to influence traits essential for fitness. Variation in condition is therefore expected to contribute to differences in reproductive investment. However, both condition and reproductive investment are complex and multi-dimensional, making them difficult to quantify comprehensively. Instead, they are typically inferred using measurable traits, which may capture only limited aspects of these constructs. Herein, we use structural equation modelling to integrate multiple proxy measurements, quantify the influence of underlying condition and reproductive investment on proxies and examine the relationship between condition and reproductive investment. Using *Gryllus vocalis* field crickets, we constructed sex-specific models to account for differences in reproductive strategies and trait expression between males and females. Both models identified proxies that were significantly influenced by condition and reproductive investment, although the strength of this influence varied across traits, suggesting that some proxies are better indicators than others. Both models also revealed significant positive relationships between condition and reproductive investment. Overall, our results demonstrate that condition positively influences reproductive investment, while revealing that commonly used proxies vary in how well they reflect these underlying constructs.

## 1. Introduction

Evolution via natural selection occurs when intraspecific variation in heritable traits differentially affects survival and reproduction. Our ability to predict evolutionary outcomes relies on accurately identifying which traits contribute to high fitness in a given environment. Individuals whose phenotypes more strongly reflect these favourable traits are often described as being in better condition than others [1–6]. An individual's condition is hypothesized to mediate the trade-offs between survival and reproduction [7,8]. Therefore, understanding the relationship between condition and reproductive investment has become key in explaining essential processes such as sexual selection [9,10], life-history evolution [7,11,12] and population dynamics [13–15]. The relationship between condition and reproductive investment impacts how selection shapes the evolution of species and our perceptions of that relationship impact how we interpret evolutionary changes over time.

Variation in how researchers conceptualize condition has contributed to the proliferation of proxy measurements, resulting in a degree of ambiguity in the components of condition that are being represented based on a researcher's chosen metric [6,8,16]. Condition has been variously estimated using body size and mass [17–19], body composition [20–24], gut fullness and feeding behaviour [25,26], blood chemistry [27,28], immune function [29,30], pheromone and hormone levels [31–34], metabolic rate [35] and gestalt or overall visual appearance [36–39]. The term ‘condition’ was originally used to describe the relative fatness of individuals [8,40] and is still frequently used in reference to an individual's available energy stores [19–24]. However, condition is also considered synonymous with Darwin's ‘vigour’ and is used interchangeably with terms like state and quality, which themselves summarize broad concepts [41–43]. Given these various conceptualizations of condition, a researcher who measures condition singularly through fatness (e.g. lipid reserves) would fail to capture the multivariate nature of condition assumed by others [44]. For this work, we define condition as an underlying trait that influences many dynamic morphological and physiological traits (i.e. those that can vary based on a combination of genetics and environment) such as body size or fat reserves.

While condition is challenging to quantify because it is an intrinsic trait that manifests through numerous extrinsic effects, approximating reproductive investment can be difficult for other reasons. Although we can measure the lifetime reproductive success of individuals, trade-offs among reproductive traits (e.g. egg size and number) and between reproductive and non-reproductive traits make it difficult to quantify the total amount of time and energy that individuals devote to reproduction. Therefore, reproductive investment, like condition, is often estimated using proxy measurements. It is indirectly quantified using traits associated with condition [6] and more directly quantified using traits such as gonad mass [45–47], competitive ability [48,49], fertility and fecundity [50,51], offspring quality [52] and offspring survival [44,51,53].

Because many theories of sexual selection are based on the idea that secondary sexual traits reveal information about an individual's underlying condition, it is necessary to develop a concrete understanding of how condition influences reproductive investment and to interpret the validity of proxies for condition and reproductive investment within that lens. Additionally, since proxies can differ between sexes—for example, male reproductive investment might be approximated by sperm count whereas female reproductive investment might use ovariole size or ovary mass—there is a need to examine the relationship between condition and reproductive investment within each sex separately. Overall, to fully understand how evolutionary forces act on individuals and populations, it is essential to clarify how condition shapes reproductive investment and refine the measures used to approximate these foundational yet elusive concepts for each sex.

To address this information gap, we used structural equation modelling (SEM) to quantify the influence of underlying condition and reproductive investment on a suite of traits often used as their respective proxies, as well as the influence of condition on reproductive investment itself. SEM encompasses both confirmatory factor analysis and path analysis and allows for the testing of complex models that include both measurement and structural components. Unlike dimensional-reduction approaches such as principal component analysis, which summarize patterns of covariation among measurable variables, SEM allows for the specification and testing of biologically motivated hypotheses about abstract and inferred traits (i.e. latent variables), making it particularly well-suited to our goals [54]. Specifically, SEM allows observed traits to be modelled as indicators of latent constructs while simultaneously evaluating the relationships among those underlying constructs. Because our models estimate a set of relationships across individuals, they characterize population-level patterns rather than individual-specific associations between the latent variables. We chose to use multiple proxies for this work because measuring singular factors in isolation (e.g. body size and total body fat for condition; development time and gonad size for reproductive investment) would fail to appropriately capture the latent and multivariate nature of condition and reproductive investment [8,55,56]. Moreover, reliance on a single proxy for a condition could lead to poor estimation of its effect on reproductive investment [6]. By explicitly modelling multiple proxies and their dependence on latent condition and reproductive investment, this approach allows us to evaluate the direct and indirect pathways that link condition to reproductive investment, providing a more biologically grounded and interpretable assessment of how condition influences reproductive investment.

Despite applications of SEM remaining sparse in scientific fields, recent studies in genetics [57,58], ecology [59] and physiology [60] have been able to use it for evaluating multiple hypothetical pathways (i.e. causal paths) between latent variables and between latent and observed variables. For this work, we capitalized on the usefulness of SEM for testing models that hypothesize causal paths between observable variables, such as whole body and gonad mass, and latent variables, such as condition and reproductive investment. Our male and female hypothesized models are built upon pre-existing knowledge of this system and are intended to explain the feasibility of our causal pathways. These models were built on the premise that if condition and reproductive investment are the underlying determinants of physiological and behavioural traits, then we will be able to integrate multiple measurable traits to determine condition and reproductive investment, thus allowing us to determine the influence of condition on reproductive investment. Both models predict that condition will be the underlying variable responsible for traits associated with organismal health and quality and informative of reproductive investment, and that reproductive investment will be the underlying variable responsible for traits associated with reproductive potential and individual fitness. Using SEM, we conducted a nuanced exploration of how condition influences reproductive investment, which should help overcome the typical ambiguity inherent in single-proxy approaches examining this relationship.

Herein, we propose two models, one for each sex, which address two key issues in our effort to understand the relationship between individual condition and reproductive investment. For each model, we assess the goodness-of-fit and interpret the influence of underlying condition and reproductive investment on their respective proxies. Additionally, we address the relationship between condition and reproductive investment within each sex-specific model. We use the vocal field cricket, *Gryllus vocalis*, as our model for this study, as males and females of the species vary in their life-history strategies and different proxies are typically used to quantify reproductive investment in each sex [61,62].

## 2. Methods

### 2.1. Study organism

*Gryllus vocalis* is found throughout much of the south-western USA [63]. This species experiences both pre- and post-copulatory sexual selection, which has led to the evolution of sex-specific traits that are predicted to signal individual potential for reproductive success.

Before mating, male field crickets compete to defend calling sites and access potential mates, resulting in selection pressures for larger body size and weaponry in the form of mandibles [48,64]. Males also possess highly specialized wing structures (i.e. harps) that are largely believed to aid in the production of acoustic signals for mate attraction [65]. While research supports the idea that females prefer males that are large and symmetrical [66], it has been proposed that harp asymmetry may confer benefits to exceptionally small males; therefore, the degree to which harp asymmetry is informed by reproductive investment could vary based on size [67]. Furthermore, female field crickets generally prefer males that invest more energy in calling, suggesting that males with increased fat stores may have an endurance benefit while performing this behaviour [68,69].

After mating, male crickets experience post-copulatory sperm competition. This process is mediated, in part, by complex accessory gland products that influence how females store sperm and fertilize their eggs [70]. Males facing higher levels of competition may have also evolved larger testes to facilitate increased sperm production, which, in combination with accessory gland products, should enhance fertilization success [71,72].

Female field crickets provision eggs based on available energy stores and those who produce more eggs or develop larger eggs may have a reproductive advantage [73,74]. Overall, females tend to allocate resources more towards reproductive tissue development, whereas males tend to invest in structural growth and energy storage [62]. As male and female field crickets differ in how they allocate resources to growth and reproduction, and have experienced different selection pressures, researchers typically quantify their reproductive investment using different proxies.

### 2.2. Experimental design

The *G. vocalis* used in this study were descended from individuals in the lab colony, which included approximately 200 breeding adults at the start of data collection for this project in August 2022. The lab colony was established with 50 individuals collected from Riverside, CA, USA, in May 2014 and supplemented with 50 additional adults collocated from the same location in May 2015.

To control for the individuals’ age in this study, cohorts were produced from eggs laid over 24 hours by five groups of parents from the laboratory colony. Hatchlings were reared in cohorts based on the parental group in 50-l plastic bins at 24°C and on a light cycle of 15 hours light : 9 hours dark. Cohort bins were provided with the same resources as the lab colony juveniles: ground alfalfa pellets and water in cotton-plugged vials, ad libitum. Egg cartons were provided as shelter. Cohort bins were checked daily for newly moulted adults to be used in our experiment.

Recommendations for determining appropriate sample sizes for SEM have shifted from universal minimum sample size rules [75]. Rather than relying on fixed thresholds, current recommendations suggest evaluating sample size requirements based on the characteristics of the proposed model. These include the number of latent variables and indicators being used, expected affected sizes, measurement quality and the potential for missing data. As will be discussed later, our male SEM included more indicator variables and was expected to have to accommodate missing data; therefore, a larger sample size was collected for use in our male model in comparison with the female model. Upon eclosion to the adult stage, 120 female and 251 male crickets were randomly selected for measurement. The selected crickets were transferred to 950 ml individual containers containing a cotton-plugged water vial, whole alfalfa pellets and egg carton. Food and water were provided ad libitum and refreshed twice weekly or more frequently as needed to prevent mould growth. Each individual was euthanized by freezing at 14 days post-eclosion, at which time they should have surpassed sexual maturity [75]. Crickets were stored frozen until post-mortem measurements were taken. All individuals were measured for total dry mass (nearest 0.1 mg), pronotum width (nearest 0.001 mm; a measure of structural body size) and dry fat mass (nearest 0.1 mg). Additional female-specific measurements included dry ovary mass (nearest 0.1 mg), average egg mass (nearest 0.01 mg) and the approximate number of eggs in the ovaries. Additional male-specific measurements included testes mass (nearest 0.1 mg), average mandible length (nearest 0.001 mm), average harp size (nearest 0.001 mm^2^) and directional asymmetry of the harps.

### 2.3. Proxies for condition

We collected measurements of several common condition proxies (i.e. indicator variables) for use in our SEMs: pronotum width, development time, fat mass and, for males, total mass. To quantify development time, cohort bins were checked daily for newly eclosed adults. Development time was calculated as the difference between the date that eggs were laid and the date of final eclosion to the adult morph. To calculate pronotum width, each individual was photographed using a Celestron MicroDirect digital microscope (Torrance, CA, USA) with a fixed focal height. The images were then analysed using ImageJ (version 1.54g) [48]. Next, to calculate the total dry mass, each cricket was dried at 55°C until a constant mass was achieved. This total mass was used as a condition indicator for the males. However, total female dry mass is strongly impacted by gonad mass, so total mass was used as a reproductive investment indicator for females [76].

To measure fat mass, each individual was placed in a cellulose filter bag following total mass measurement. Crude fat was extracted via Soxhlet reflux for 6 hours using petroleum ether (Fisher Scientific, Hanover Park, IL, USA). Each sample was then dried again at 55°C until a constant mass was achieved. Fat mass was obtained by calculating the difference between lean dry mass (after extraction) and total dry mass (before extraction).

### 2.4. Proxies for female reproductive investment

In addition to our condition indicator variables, we collected several reproductive investment indicators for females: ovary mass, number of eggs within the ovaries and total mass. To obtain the ovary mass and number of eggs in the ovaries, the gonads were dissected and weighed. Next, 30 eggs were randomly selected from the ovaries and weighed together to provide an estimate of the average egg mass. The approximate number of eggs in the ovaries was calculated by dividing the wet mass of the ovaries by the average wet egg mass. These eggs were then returned to the ovary masses and dried at 55°C until a constant mass was achieved to obtain the ovary mass. Total mass was obtained in the manner previously described for males.

### 2.5. Hypothetical female model

Our hypothetical female SEM (figure 1*a*) included our condition and reproductive investment indicator variables and incorporated relationships between total mass and fat mass [77] since they have been shown to be highly correlated in other cricket species, and ovary mass and number of eggs as they are also expected to be correlated [78]. We used pronotum width, a measurement of structural body size, as our reference variable for condition [77]. Additionally, we used ovary mass as the reference variable for reproductive investment as it has been shown to be a strong predictor in past studies [78].

**Figure 1.**
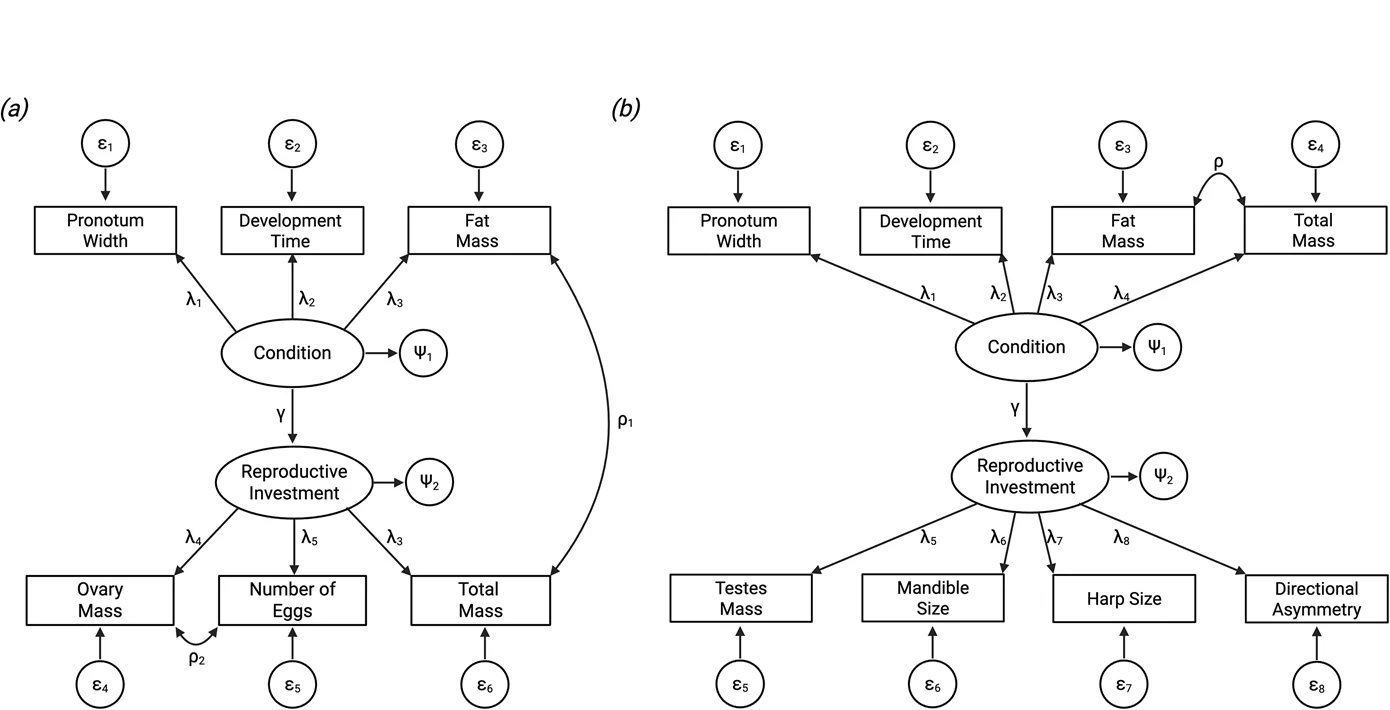
Hypothetical (*a*) female and (*b*) male structural equation models showing proposed relationships between latent (ovals) and indicator (rectangles) variables. Circles represent error terms. Single-headed arrows indicate causal paths, and double-headed arrows represent correlations between indicator variables. Lambda (λ) and gamma (γ) denote path coefficients.

### 2.6. Proxies for male reproductive investment

We collected data on four indicator variables for reproductive investment in our male SEM: testes mass, mandible length, harp size and harp directional asymmetry. To determine the testes mass, we dissected the gonads of each male and dried them, as previously described. To obtain the average mandible length, we dissected and photographed the mandibles of each male using a Celestron MicroDirect digital microscope with a fixed focal height and analysed the images using ImageJ (version 1.54g) [48]. In cases where one mandible was damaged, the length of the intact mandible was used in place of average length. In cases where both mandibles were damaged, the mandible size was not included in the dataset. To measure the average harp size and directional asymmetry of the harps, the wings of each male were also dissected and photographed using a digital microscope and analysed using the same software as the mandibles [66]. Directional asymmetry was calculated as the difference in size between the left and right harps. As with the mandibles, in cases where one wing was damaged in such a way that the harp size could not be measured, the single intact harp size was used in place of the average size and harp asymmetry was not included in the dataset. If both wings were damaged, neither harp size nor harp asymmetry was included.

### 2.7. Hypothetical male model

Our hypothetical male SEM (figure 1*b*) included all the previously mentioned condition and reproductive investment proxies. Similar to the female model, we included a relationship between total dry mass and dry fat mass and chose to use pronotum width as our reference variable for condition. However, for our male SEM, we selected testes mass as our reference variable for reproductive investment, as it influences the amount and speed at which sperm can be produced, impacting sperm competition success [79].

### 2.8. Model fitting and evaluation

To be included in the dataset for model fitting, the crickets needed to reach sexual maturity. During dissections, three female crickets were found to have underdeveloped ovaries and two male crickets had underdeveloped testes, suggesting that they were not sexually mature by the time they were euthanized. Additionally, six female and eight male crickets died before day 14 post-eclosion and were, therefore, excluded from use in their respective models. Therefore, we used data collected from 111 females and 241 males to fit the final models.

To ensure the validity of our analyses, we assessed both the univariate and multivariate normality of the data. Univariate normality was evaluated using Anderson–Darling tests [80] and multivariate normality was assessed using Henze–Zirkler tests [81], which revealed significant deviations in normality for both female and male datasets (*p* < 0.05). Given these deviations, we used the Huber–White robust standard error estimator with robust maximum likelihood (MLR) when fitting the models [82,83]. For cases with missing data, we used full information maximum likelihood (FIML), which allows for parameter estimation under a missing at random (MAR) assumption using all available data. All variables were examined for sufficient coverage before inclusion in the model. Then the models were each defined, fitted and analysed using the *lavaan* package [84] in R (Version 4.4.1).

While the chi-squared test is typically used to determine model fit, its sensitivity is based on sample size and model complexity, making it a poor choice for the evaluation of our models [85,86]. Therefore, we examined model fit using the robust comparative fit index (CFI.robust) and the robust Tucker–Lewis index (TLI.robust) to determine the incremental fit of the models relative to baseline models, as they are less sensitive to sample size. Additionally, we used the standardized root mean square residual (SRMR) to assess absolute model fit to the data [87].

## 3. Results

### 3.1. Female model

We fit and examined our hypothesized model, which predicted several potential indicator variables for condition and reproductive investment, and that condition indicates reproductive investment. Our model (figure 2) generally showed an acceptable fit based on the CFI.robust, TLI.robust and SRMR results (table 1).

**Figure 2.**
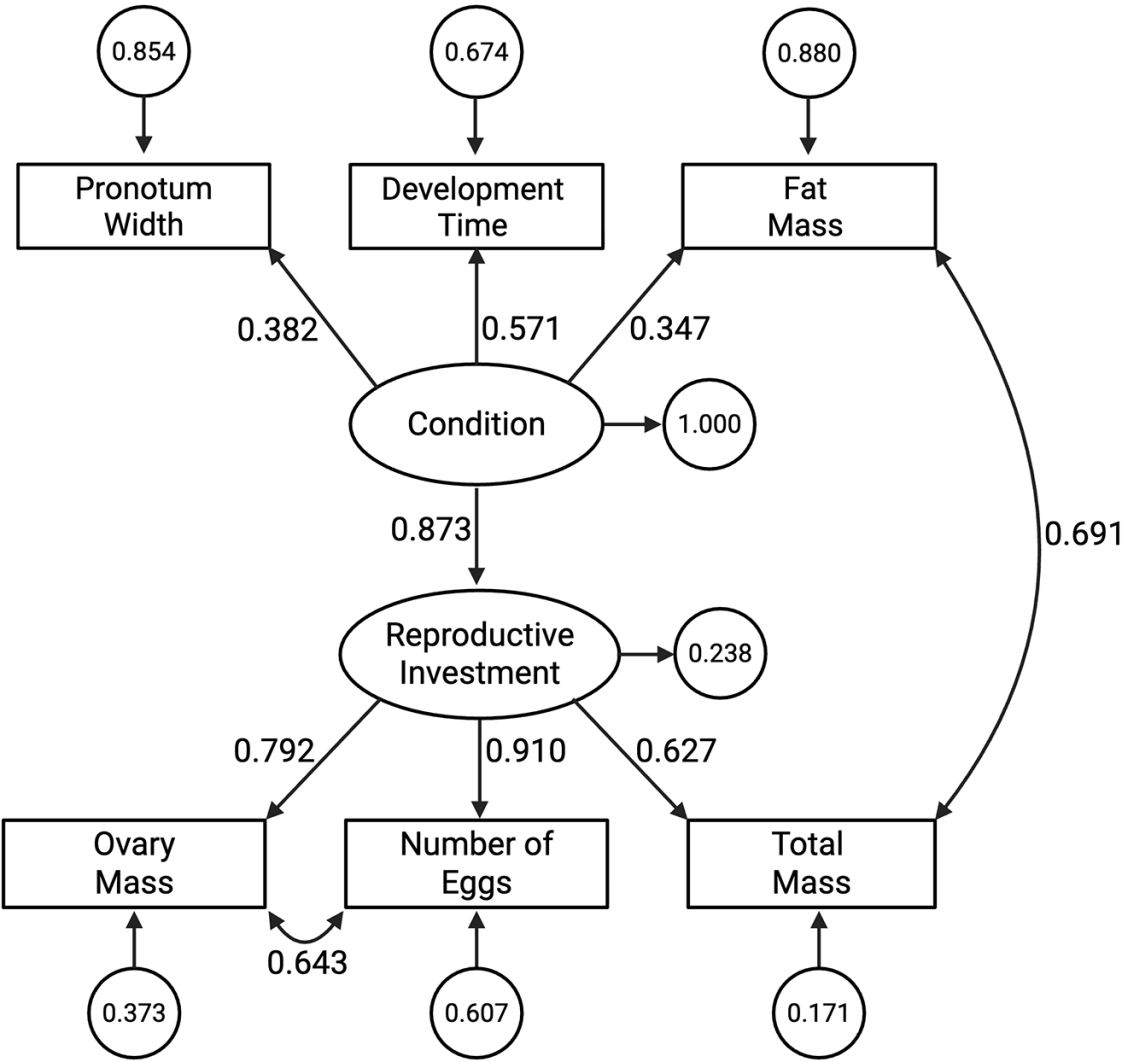
Final standardized female SEM depicting the significant relationships between latent condition and reproductive investment, along with their observed indicators, factor loadings, error terms, the regression coefficient for the condition-reproductive investment path, the variance for reproductive investment, and covariances.

**Table 1.** Results of model fit for the female and male models, alongside recommendations for acceptable fit [87]. The scaled robust comparative fit index (CFI.robust), robust Tucker–Lewis index (TLI.robust) and standardized root mean squared (SRMR) results are included.

fit index	acceptable fit	female model	male model
CFI.robust	≥ 0.90	0.97	0.93
TLI.robust	≥ 0.90	0.93	0.89
SRMR	≤ 0.08	0.05	0.07

All factor loadings, standardized using all path information (std.all), for both condition and reproductive investment, were significant (*p* < 0.05), supporting the validity of the hypothesized model (supplementary material, table S1). For condition, the loadings ranged from 0.347 to 0.571, and for reproductive investment, they ranged from 0.627 to 0.910. These findings suggest that all indicator variables meaningfully reflect their latent constructs, with development time most strongly representing condition (std.all = 0.571; *p* = 0.003) and total mass most strongly representing reproductive investment (std.all = 0.910; *p* < 0.001). The regression path from condition to reproductive investment was positive and significant (std.all = 0.873; *p* = 0.010), indicating that individuals in better condition tended to have higher reproductive investment. Most of the covariances and variances among the observed variables were significant. The residual covariances between fat mass and total mass (std.all = 0.691; *p* = 0.029) and ovary mass and number of eggs (std.all = 0.643; *p* < 0.001) were significant. Among the variances, most observed variables showed significant unexplained variability. However, the residual variances for total mass (std.all = 0.171; *p* = 0.347), condition (std.all = 1.000; *p* = 0.070) and reproductive investment (std.all = 0.238; *p* = 0.141) were not statistically significant, indicating that a substantial portion of the variance was accounted for by the model.

### 3.2. Male model

As with the female model, we fit and examined our hypothesized male model, which predicted several potential indicator variables for condition and reproductive investment, and that condition indicated reproductive investment. Similar to our female model, the male model (figure 3) also generally showed an acceptable fit to the data based on our evaluation metrics (table 1).

**Figure 3.**
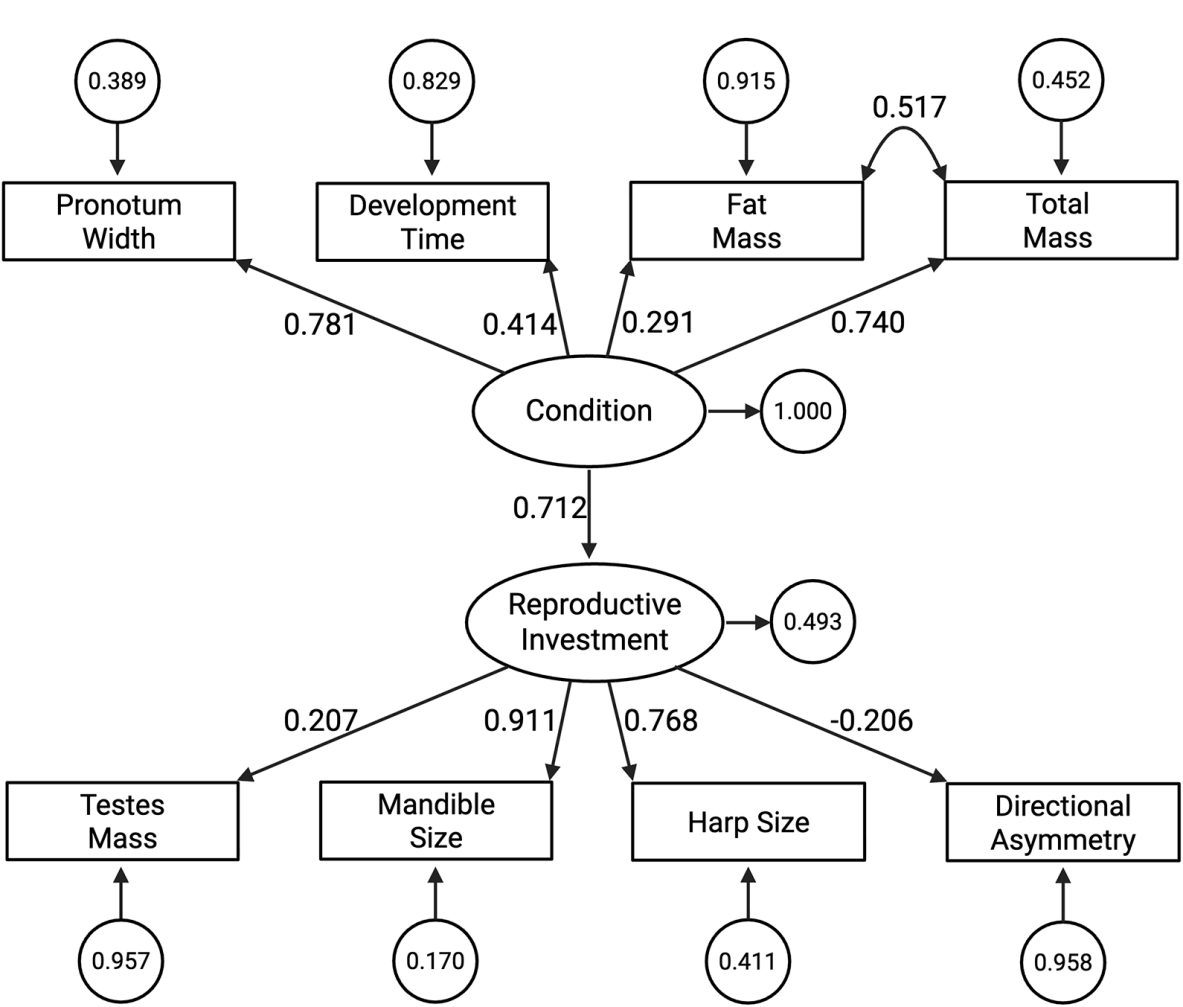
Final standardized male SEM depicting the significant relationships between latent condition and reproductive investment, along with their observed indicators, factor loadings, error terms, the regression coefficient for the condition-reproductive investment path, the variance for reproductive investment, and covariance between fat and total mass.

All factor loadings for both condition and reproductive investment were significant (*p* < 0.05), with the exception of directional asymmetry (*p* = 0.056; supplementary material, table S2). These results generally support the validity of our hypothesized model. For condition, the standardized loadings ranged from 0.291 to 0.781. For reproductive investment, the standardized loadings ranged from −0.206 to 0.911. Pronotum width contributed most strongly to the latent construct of condition (std.all = 0.781). Mandible size contributed most strongly to the latent construct of reproductive investment (std.all = 0.911; *p* = 0.002). The regression path from condition to reproductive investment was positive and significant (std.all = 0.712; *p* = 0.005), indicating that, similar to females, males in better condition tended to have higher reproductive investment. Notably, the residual covariance between fat mass and total mass was significant (std.all = 0.517; *p* = 0.023). Among the variances, most observed variables showed significant unexplained variability. However, the residual variances for mandible size (std.all = 0.170; *p* = 0.368) and reproductive investment (std.all = 0.493; *p* = 0.117) were not statistically significant, indicating that a substantial portion of their variance was accounted for by the model.

## 4. Discussion

Using a SEM framework, we modelled condition and reproductive investment as latent, multivariate traits in male and female *G. vocalis* crickets. In both models, condition significantly and positively informed reproductive investment and multiple indicator variables significantly loaded onto their respective latent constructs. These loadings represent the extent to which individual proxies reflect variation in the underlying latent trait and are best interpreted relative to other indicators within the same biological context. The relative strength of the loadings in our models varied considerably between sexes and across indicators. Taken together, these findings demonstrate that condition and reproductive investment cannot be fully captured by any single morphological variable and that many indicators likely depend on sex-specific life-history strategies.

### 4.1. The influence of condition on indicator variables

Both the male and female models used development time, pronotum width and fat mass to approximate condition. Additionally, in the male model, total mass was included as an indicator for condition. In literature, researchers will often use one of these variables as a proxy for condition, and many assume that each trait captures the condition of an individual to a similar degree. While our models support a significant influence of condition on all included indicators, the magnitude of those loadings varied widely. This suggests that different indicators reflect distinct and only partially overlapping aspects of condition, and none fully capture it. Consequently, studies relying on a single proxy, or assuming equivalence among proxies, risk drawing incomplete or biased conclusions. For example, in studies of condition-dependent traits, a weak association between the trait of interest and a single proxy for condition could lead researchers to conclude that the trait is not condition dependent. However, the relative value of this trait may depend on a combination of other traits, some of which may be dependent on condition.

For the female model, development time was found to be the strongest indicator for condition, although the strength of the loading was moderate (std.all = 0.571). Similarly, development time was shown to be a moderate indicator of condition in males (std.all = 0.414). Overall, this suggests a positive association between development time and the latent variable of condition. This is consistent with previous studies showing that *G. vocalis* with longer development times attain larger adult body sizes and possess better phenoloxidase-based immunity [61]. Therefore, development time appears to reflect somatic investment during growth, potentially owing to prolonged resource acquisition and physiological allocation. However, the moderate loadings in both models indicate that development time only captures a part of overall condition. Because development time and adult body size are central to many models of life-history trade-offs, this finding has broader implications. While prolonged development can confer advantages in terms of size and immunity, it can simultaneously impose costs, such as delayed reproduction or increased juvenile mortality [88]. Thus, the extent to which development time reflects condition should depend on which side of this trade-off most strongly influences an individual's reproductive potential in its given environmental context. Studies that rely on development time as a primary proxy for condition may under-represent other dimensions of condition that are uncoupled from growth rate.

Pronotum width was also a significant indicator for condition in both models, though its relative strength differed markedly based on sex. For the male model, pronotum width loaded most strongly onto condition (std.all = 0.781), whereas it showed a low to moderate association (std.all = 0.382) in the female model. As a measure of fixed adult body size, pronotum width reflects the cumulative outcome of growth and resource acquisition during development rather than the speed of development. In resource-rich environments, such as laboratory settings where food is provided ad libitum, larger size can be achieved without sacrificing other aspects of health, such as immune function or energy storage. For males, being large confers an advantage during male-male combat and is associated with having larger mandibles and harps, traits that offer reproductive benefits [48,67]. Because substantial resource investment is required to develop and maintain such structures, large structural body size should serve as a relatively honest measure of male condition.

By contrast, the weaker association found in the female model implies that structural body size reflects underlying condition to a lesser degree. As females of this species do not typically engage in intrasexual combat, the same pressure as that on males for a large structural body size should not exist. However, high resource availability during development should still result in a larger body size, which is broadly associated with better health, regardless of sex [61]. This likely explains why pronotum width only modestly reflects condition in the female model. Taken together, the differences between the male and female models highlight an important limitation of metrics related to structural body size. Although these traits are convenient to measure and highly repeatable, structural size reflects the cumulative outcome of resource acquisition and allocation during development rather than the present condition of an individual. Consequently, body size alone may not capture all dimensions of condition, particularly in systems where multiple developmental pathways can produce similar adult body sizes. Several size-corrected condition indices, such as the scaled mass index, have been developed to account for this limitation by incorporating both structural size and body mass [4]. While such approaches may provide a more refined estimate of condition than structural size alone, they still represent a single composite proxy and may not capture all dimensions of condition.

Fat mass was a statistically significant indicator for condition in both the male and female models; however, the loading strength was the lowest among all indicators (female std.all = 0.347; male std.all = 0.291). Lipid stores are commonly used as proxies for condition across taxa because they reflect the energy available for growth and reproduction [20–22]. However, the modest loadings observed in both models suggest that, surprisingly, energetic reserves might only reflect a small component of condition. Body fat levels can be sensitive to short-term environmental variation or changes in activity level, so it is possible that recent feeding history, lifetime variation in food availability and performance could lead to changes in fat mass, making it a less reliable indicator of condition [89–92]. Consequently, this proxy might be less stable than some other variables, such as pronotum width, which is fixed at adulthood in crickets. Furthermore, reliance on fat mass in isolation could underestimate long-term developmental or physiological investments related to condition.

The inclusion of residual covariances between fat mass and total mass in both models reflects the expectation that these two proxies share variance beyond what would be informed by condition, as total mass includes the mass of energetic reserves. We found significant covariances between fat mass and total mass in both models. Furthermore, total mass was found to be a strong and significant indicator for condition in our male model (std.all = 0.740; *p* < 0.001), probably because total mass reflects energy reserves, muscle mass and overall body size. Total mass should capture how variation in condition may benefit males during combat and signalling, similar to how structural body size would [93,94]. Taken together, the lower strength of fat mass as an indicator in both models, the higher strength of total mass as a male indicator for condition, and the covariance between these two proxies, suggest that the energetic and structural components of condition are intertwined but not interchangeable.

Overall, variation in loading strength across condition indicators reinforces condition's multivariate nature and how it is shaped by sex-specific allocation strategies. Therefore, researchers should avoid treating condition proxies as equivalent and, when possible, measure multiple indicators to more fully capture how environmental experiences impact individuals.

### 4.2. The influence of reproductive investment on female model indicator variables

Female reproductive investment is often quantified using measurements indicative of fecundity and fertility. Our female model incorporated ovary mass, number of developed eggs contained within the ovaries and total mass to approximate reproductive investment.

Ovary mass, a direct measure of reproductive tissue, was a relatively strong and significant indicator (std.all = 0.792), supporting its widespread use as a proxy for female reproductive investment. Number of eggs was also a significant but more moderate indicator of reproductive investment (std.all = 0.627), suggesting that egg count alone cannot fully capture an individual's reproductive investment. Because female field crickets can continuously acquire resources and provision eggs throughout adulthood, the number of developed eggs contained within the ovaries at any single time point may not be predictive of lifetime reproductive potential [95,96]. The significant residual covariance between ovary mass and egg number reflects that both these variables capture some overlapping, but not identical, degree of reproductive investment.

Of all the female indicators for reproductive potential, total mass was the strongest (std.all = 0.910), likely because overall body mass integrates several components relevant to reproductive investment, notably reproductive tissue mass and energetic reserves, which can be allocated to egg production and other reproductive behaviours. As females of this species invest heavily in reproductive organs, it is reasonable to assume that this would influence the overall mass of individuals [62]. However, in other systems where reproductive tissue and energy reserves contribute less to overall mass, it could be worth exploring how this measurement reflects condition as opposed to reproductive investment.

### 4.3. The influence of reproductive investment on male model indicator variables

Male reproductive success largely depends on access to and acquisition of mates [97]. Thus, traits associated with male-male combat and signalling to potential mates may serve as better indicators of reproductive investment in males than measuring gonad mass alone. Therefore, we included three indicators associated with mate attraction and acquisition in our male model (i.e. mandible size, harp size and harp directional asymmetry), in addition to testes mass.

Gonad mass is a relatively common proxy for reproductive investment across taxa [45–47] and ovary mass was shown to be a markedly strong indicator in our female model. However, testes mass was found to be a significant but comparatively weak indicator (std.all = 0.207) for reproductive investment in our male model. Past work has shown that male *G. vocalis* tend to allocate more energy and resources towards somatic growth than reproductive tissue in comparison with females [62], so in combination with our model results, it seems that reproductive tissue mass is not a reliably strong indicator of male reproductive investment. Mandible size, however, is often a predictor of male-male competition success in crickets and other insects [48,98] and was the strongest indicator of reproductive investment in the male model (std.all = 0.911). In essence, males with larger weaponry should have an advantage in competitive interactions to access mates. The magnitude of the loading here, in contrast to testes mass, suggests that reproductive investment in males is more strongly tied to pre-copulatory competition for mates than to post-copulatory competition for fertilization success.

Harp size was also a relatively strong and significant indicator for male reproductive investment (std.all = 0.768). This is unsurprising, given the abundance of literature supporting harp size as a key sexually selected trait in crickets [66,67,99]. Interestingly, harp directional asymmetry was a weak, non-significant indicator for reproductive investment (std.all = −0.206; *p* = 0.056). Directional asymmetry has long been debated as an indicator of developmental stability, individual health and potential reproductive success [100–102]. However, some studies on crickets have suggested that males may benefit from directional asymmetry as a way to compensate for smaller body size [67]. Taken with the results of our model, this suggests that in some cases, possibly when all males are of large body size and high condition, symmetrical harps might better reflect reproductive investment, but if males are small, asymmetry might be beneficial for reproductive success and reflect an alternative tactic for reproductive investment, therefore making harp asymmetry a context-specific indicator and not widely applicable for predicting reproductive investment on its own.

Altogether, these findings highlight that the biological relevance of proxies for reproductive investment depends on the pathways through which reproductive success is achieved in each sex. Even when analogous measures, such as gonad mass, are collected in both males and females, they may differ substantially in how well they reflect reproductive investment. Therefore, proxy selection should be guided by the reproductive biology of the study system of interest rather than by the availability of equivalent measurements across sexes.

### 4.4. The influence of condition on reproductive investment

Both our female and male models showed an acceptable overall fit and a significant positive relationship between condition and reproductive investment. The non-significant residual variances for reproductive investment in both the female (*p* = 0.141) and male (*p* = 0.117) models indicate that a substantial portion of the variance was accounted for by the hypothesized pathways. These findings are consistent with studies of life history that demonstrate how condition is tightly linked to resource availability and acquisition through both development and adulthood, eventually allowing for higher reproductive investment [88,103–107]. Favourable environmental conditions throughout an individual's lifetime should provide a pool of resources that can be partitioned among maintenance, growth and reproduction, without significant trade-offs. Because reproduction is energetically costly, individuals in higher condition should be able to invest more in reproductive traits, leading to higher reproductive success without compromising their own growth and survival.

Our models reflect the relationship between condition and reproductive investment for our population of individuals. Although individuals in higher condition should have the capacity for higher reproductive investment, life-history theory predicts that individuals may vary in both their abilities to acquire resources and in how they allocate those resources among maintenance, growth and reproduction. Such heterogeneity in allocation strategies is not explicitly represented by our framework and, therefore, cannot be distinguished from other sources of unexplained variation in the models. Future studies could expand upon our approach to evaluate how the relationship between condition and reproductive investment differs among individuals.

Overall, the positive association between condition and reproductive investment in both our male and female models suggests that, on average, higher condition reflects a higher capacity for resource acquisition and utilization. For females, higher condition allows resources to be translated into increased reproductive tissue and egg provisioning. In males, higher condition enables an investment in costly sexually selected traits that are critical for mate acquisition. Thus, despite sex-specific patterns for optimizing reproductive success, the positive association of condition with reproductive investment reflects a shared energetic constraint, where both males and females with greater resource acquisition and partitioning capacity can afford higher reproductive investment.

Taken together, our results demonstrate the value of explicitly modelling condition and reproductive investment as latent variables informed by multiple indicators. SEM provides a powerful framework for disentangling how measurable traits reflect the underlying traits of individuals. Broadly, our findings highlight the importance of aligning proxy choice with the biology of study organisms and being selective in which components of condition and reproductive investment one is investigating. Although the specific proxies identified here are unlikely to be generalized across taxa, the analytical framework is broadly applicable. Rather than relying on a single conventional proxy, researchers should identify multiple candidate indicators based on the natural history and life-history strategies of their study organisms and evaluate their relative loadings on latent constructs. This approach provides a means of testing assumptions about commonly used proxies while allowing measurement models to be refined as biological knowledge of a system improves. Moving forward, adopting multivariate approaches should improve our ability to interpret condition-dependent traits, clarify sex-specific life-history trade-offs and generate more robust inferences about the mechanisms linking condition and reproductive investment.

## Supplementary Material



## Data Availability

All data and code used in the development of these models are contained within the supplementary material [108].
