# Supplementary material for: Latent variable modelling of condition and reproductive investment in Gryllus vocalis field crickets
Source: R Soc Open Sci. 2026 Oct 28;13(10):261085. doi: 10.1098/rsos.261085 (PMC13714378; doi:10.1098/rsos.261085)
Supplement: Supplementary file 1 [file rsos.261085.zip › rsos.261085/rsos261085_si_001.docx]

Supplemental Table 1. Raw parameter estimates (Estimate), standard errors (Std. Err.), z-values, P-values, standardized estimates with respect to the latent variable variance (Std.lv), and fully standardized estimates (Std.all) for our female SEM. Included are the factor loadings between latent and indicator variables, the regression between latent variables, covariances, and variances.

|  | Estimate | Std.Err. | z-value | *P*-value | Std.lv | Std.all |
| --- | --- | --- | --- | --- | --- | --- |
| **Latent Variables** |  |  |  |  |  |  |
| Condition ~  Pronotum Width | 1.000 |  |  |  | 0.523 | 0.382 |
| Condition ~  Development Time | 22.401 | 7.598 | 2.948 | 0.003 | 11.726 | 0.571 |
| Condition ~  Fat Mass | 11.050 | 3.922 | 2.817 | 0.005 | 5.784 | 0.347 |
| Reproductive Investment ~  Ovary Mass | 1.000 |  |  |  | 21.489 | 0.792 |
| Reproductive Investment ~  Total Mass | 1.784 | 0.314 | 5.674 | <0.001 | 38.343 | 0.910 |
| Reproductive Investment ~  Number of Eggs | 3.418 | 0.380 | 8.991 | <0.001 | 73.443 | 0.627 |
| **Regressions** |  |  |  |  |  |  |
| Reproductive Investment ~  Condition | 35.829 | 13.920 | 2.574 | 0.010 | 0.873 | 0.873 |
| **Covariances** |  |  |  |  |  |  |
| Fat Mass ~  Total Mass | 188.352 | 86.153 | 2.186 | 0.029 | 188.352 | 0.691 |
| Ovary Mass ~  Number of Eggs | 971.132 | 263.346 | 3.688 | <0.001 | 971.132 | 0.643 |
| **Variances** |  |  |  |  |  |  |
| Pronotum Width | 1.606 | 0.495 | 3.247 | 0.001 | 1.606 | 0.854 |
| Development Time | 284.750 | 49.608 | 5.740 | <0.001 | 284.750 | 0.674 |
| Fat Mass | 244.340 | 47.386 | 5.156 | <0.001 | 244.340 | 0.880 |
| Ovary Mass | 274.336 | 75.287 | 3.644 | <0.001 | 274.336 | 0.373 |
| Total Mass | 303.857 | 323.281 | 0.940 | 0.347 | 303.857 | 0.171 |
| Number of Eggs | 8317.388 | 1219.462 | 6.821 | <0.001 | 8317.388 | 0.607 |
| Condition | 0.274 | 0.151 | 1.814 | 0.070 | 1.000 | 1.000 |
| Reproductive Investment | 109.998 | 74.793 | 1.471 | 0.141 | 0.238 | 0.238 |

Supplemental Table 2. Raw parameter estimates (Estimate), standard errors (Std. Err.), z-values, P-values, standardized estimates with respect to the latent variable variance (Std.lv), and fully standardized estimates (Std.all) for our male SEM. Included are the factor loadings between latent and indicator variables, the regression between latent variables, covariances, and variances.

|  | Estimate | Std. Err. | z-value | *P*-value | Std.lv | Std.all |
| --- | --- | --- | --- | --- | --- | --- |
| **Latent Variables** |  |  |  |  |  |  |
| Condition ~  Pronotum Width | 1.000 |  |  |  | 0.839 | 0.781 |
| Condition ~  Development Time | 10.103 | 1.486 | 6.798 | <0.001 | 8.474 | 0.414 |
| Condition ~  Fat Mass | 7.329 | 1.794 | 4.084 | <0.001 | 6.147 | 0.291 |
| Condition ~  Total Mass | 30.347 | 2.552 | 11.892 | <0.001 | 25.452 | 0.740 |
| Reproductive Investment ~  Testes Mass | 1.000 |  |  |  | 0.396 | 0.207 |
| Reproductive Investment ~  Mandible Size | 1.098 | 0.355 | 3.094 | 0.002 | 0.435 | 0.911 |
| Reproductive Investment ~  Harp Size | 4.826 | 1.661 | 2.906 | 0.004 | 1.913 | 0.768 |
| Reproductive Investment ~  Directional Asymmetry of Harps | -0.310 | 0.162 | -1.912 | 0.056 | -0.123 | -0.206 |
| **Regressions** |  |  |  |  |  |  |
| Reproductive Investment ~  Condition | 0.337 | 0.121 | 2.785 | 0.005 | 0.712 | 0.712 |
| **Covariances** |  |  |  |  |  |  |
| Fat Mass ~  Total Mass | 241.090 | 106.084 | 2.273 | 0.023 | 241.090 | 0.517 |
| **Variances** |  |  |  |  |  |  |
| Pronotum Width | 0.449 | 0.188 | 2.381 | 0.017 | 0.449 | 0.389 |
| Development Time | 348.140 | 27.698 | 12.569 | <0.001 | 348.140 | 0.829 |
| Fat Mass | 407.213 | 101.375 | 4.017 | <0.001 | 407.213 | 0.915 |
| Total Mass | 533.971 | 158.343 | 3.372 | 0.001 | 533.971 | 0.452 |
| Testes Mass | 3.519 | 0.547 | 6.430 | <0.001 | 3.519 | 0.957 |
| Mandible Size | 0.039 | 0.043 | 0.900 | 0.368 | 0.039 | 0.170 |
| Harp Size | 2.549 | 0.327 | 7.808 | <0.001 | 2.549 | 0.411 |
| Directional Asymmetry of Harps | 0.342 | 0.076 | 4.495 | <0.001 | 0.342 | 0.958 |
| Condition | 0.703 | 0.087 | 8.097 | <0.001 | 1.000 | 1.000 |
| Reproductive Investment | 0.077 | 0.049 | 1.566 | 0.117 | 0.493 | 0.493 |
